# The relationship between motor development and social adaptability in autism spectrum disorder

**DOI:** 10.3389/fpsyt.2022.1044848

**Published:** 2022-11-23

**Authors:** YanJie Chen, Xi Fei, TianChen Wu, HongJuan Li, NiNa Xiong, RuiYun Shen, Ying Wang, AiMin Liang, Huan Wang

**Affiliations:** ^1^Department of Children’s Health Care Center, Beijing Children’s Hospital, Capital Medical University, National Center for Children’s Health, Beijing, China; ^2^School of Sports Science, Beijing Sport University, Beijing, China; ^3^Department of Obstetrics and Gynecology, Peking University Third Hospital, Beijing, China; ^4^China Institute of Sport Science, Beijing, China

**Keywords:** autism, motor development, gross motor, fine motor, social adaptability

## Abstract

**Objectives:**

Autism spectrum disorders(ASD)describe a wide range of pervasive developmental disorders by core symptoms including deficits in social communication and interaction, as well as restricted, repetitive, and stereotyped behaviors. At the same time, some children with autism are accompanied by motor development disorder. Many studies have confirmed that the motor development impairment was significantly associated with the social problems associated with ASD. Thus, this study aimed to investigate how motor development affects social adaptability in children with ASD to provide references for early ASD intervention.

**Materials and methods:**

The case data of children’s health care were selected in 2021. Motor development was assessed with the Developmental Behavior Assessment Scale for Children Aged 0–6 years. Social adaptability was measured using the Japanese S-M Social Living Skills Scale. Statistical analysis was conducted with SPSS 22.0 software package. Data were analyzed using independent samples *t*-test and logistic regression.

**Results:**

A total of 198 cases comprising 140 boys (70.71%) and 58 girls (29.29%) were included, and the average age of participants was 3.40 ± 1.06 years, with 3.33 ± 1.18 years in the typical development (TD) children group and 3.46 ± 0.95 years in the ASD group. The social adaptability of 107 ASD children was abnormal, including 37 children (34.5%) with marginal, 48 children (44.9%) with mild, 17 children (15.9%) with moderate, and 5 children (4.7%) with severe. In 91 TD children, there were 51 children (56.04%) with normal social adaptability, 38 children (41.75%) with marginal, 2 children (2.19%) with mild, and nobody with moderate or severe. The ASD children had lower levels of developmental behavior than those of TD children, and the difference was statistically significant. The results of logistic regression showed that fine motor increased by 1 unit, and the OR value of one level decreased in social adaptability was 2.24 times (OR = e^0^.^807^ = 2.24).

**Conclusion:**

In children with ASD, not only motor development is delayed, but also social adaptability is affected, and fine motor skill may be important for social adaptability.

## Introduction

Autism spectrum disorder (ASD) comprises a group of neurodevelopmental disorders characterized by persistent deficits in social communication and interaction and the presence of restricted, repetitive behaviors (1). Its population has dramatically grown worldwide (2) with 78 million people diagnosed with ASD (3), entailing remarkably medical costs and social hardships. As a result, autism is now a major public health issue that seriously affects the health of children.

In the 1980s, the concept of cerebellar dysfunction was introduced to explain the link between movement disorders and other neurodevelopmental disorders (4, 5). In the last four decades of research, in addition to typical ASD behaviors, children with ASD show varying degrees of impairment in motor development (6–8). Compared with typically developing (TD) children, children with ASD often have difficulties with motor development (9), such as coordination disorders in gross and fine activities, poor balance, and movement posture instability (10–12). Motor is an individual’s “somatic-psychological” response to the environment under the multi-level regulation of the brain. It is not only the basic means of effective interaction between the individual and the environment, but also an important window to observe and detect the physical and mental development of the individual (13). Motor development occurs throughout the course of a person’s life and is closely related to the development of cognitive abilities, language, emotions, and social adaptability (10). Good motor development in early childhood will have positive effects on cognitive, language, emotional and social development, and provide favorable conditions for the overall development of individuals in the future. On the other hand, if the motor development is obstacle, the individual development will also be hindered (14–17). Motor development disorder is a non-verbal neuropsychological dysfunction that brings difficulties to the individual’s daily life and causes cognitive deficits and social interaction limitations (18).

Social adaptability refers to an individual’s ability to independently handle daily life and take social responsibility to the extent expected by his age and social and cultural conditions (19), including self-care, labor skills, language development, social responsibility, and so on. The social dysfunction of autistic patients leads to low social adaptability. Several studies have demonstrated that motor performance plays a significant role in cognitive and social functioning (20–23). When a child is able to crawl or walk, he or she can explore the environment more in novel ways, which contributes to their cognitive and social functioning. Motor development and social functioning are linked at the neurobiological level. Poor action ability makes children unable to participate in peer games and even weaken their social adaptability in the long run. Amygdala and prefrontal cortex (PFC) share a reciprocal connection and both play a crucial role in social behavior and motor planning (24). In autism spectrum disorders, this close relationship between motor, cognitive, and social development is also evident (25). At present, there are many studies on the social problems of autism. At the same time, many studies have found that children with autism have poor motor development. However, is there a relationship between the motor domain and social adaptability, and which type of motor skill is more closely related to social adaptability? Therefore, this study aimed to understand the characteristics of motor development and social adaptability in children with ASD, explore the relationship between motor development and social adaptability in children with ASD, in order to provide reference for early intervention of ASD.

## Materials and methods

### Participants

The case data of children’s health care were selected in 2021. The case group was composed of 107 children who were clearly diagnosed with autism in the outpatient department. ASD diagnosis was performed according to DSM-5 clinical criteria. Meanwhile, 97 TD children were chosen from daily physical examination. Inclusion criteria: (1) diagnosed by a health care physician of our hospital; (2) complete the developmental behavior and social adaptability assessment; And (3) without training intervention. Exclusion criteria: (1) other severe psychiatric and neurological diseases, known chromosomal diseases, inherited metabolic diseases, autoimmune diseases, other major physical diseases, such as heart, liver, renal insufficiency, epilepsy, audiovisual disorders, severe traumatic brain injury. (2) incomplete data. If the same child was measured multiple times, the first test data was selected. This study was approved by the Ethics Committee of Beijing Children’s Hospital, Capital Medical University (IEC-C-008-A08-V.05.2).

### Measurements

Developmental behavior was evaluated by the Developmental Behavior Assessment Scale for Children Aged 0–6 years which released by the National Health and Family Planning Commission, PRC in 2017. The scale includes 211 items on several areas. This technique was used to evaluate the children’s (1) language, (2) adaptability, (3) fine motor skills, (4) social ability, (5) gross motor skills, and (6) developmental quotient (DQ). The gross motor refers to the basic movement skills related to walk, run, and jump shot. The fine motor refers to the basic motor abilities related to hand function. The developmental quotient reference range is 130 as excellent, 100–129 as good, 99–80 as moderate, and 70–79 as critically low. Scores under 70 indicate mental retardation. The *test*-*retest* reliability and criterion validity of the scale were 0.73–0.81 and 0.95 respectively (26).

Social adaptability was measured using the Normal Development of Social Skills from Infant to Junior High School Children(S-M). The scale includes 132 items on several areas and evaluates from two aspects: the degree of individual independence, the degree of meeting personal and social obligations and requirements. The scale was filled out by parents or daily caregivers according to the children’s corresponding ages. Higher scores on this scale indicate less social adjustment, and lower scores indicate greater social adjustment. Standard score ≥ 10 is considered as normal, 9 as marginal, 8 as mild, 7 as moderate and under 6 as severe. The scale is the Chinese adaptation and revision of the original test: the validity and *test*-*retest* reliability have been validated (validity: 0.95, *test*-*retest* reliability: 0.98) (26).

In the actual evaluation, all of the evaluation personnel are hospital staff who underwent professional training to obtain the corresponding qualifications.

### Statistical methods

Statistical analysis was conducted with SPSS 22.0 software package. Continuous data consistent with normal distribution were demonstrated by mean ± standard deviation (SD). Categorical data will be presented in terms of frequencies or percentages. An independent samples *t*-test was used to compare the developmental behavior of ASD and TD children. Logistic regression analysis was conducted to analyze the relationship between motor development of ASD children and social adaptability. *P* < 0.05 was considered statistically significant.

## Results

### General information of the research objects

A total of 198 cases comprising 140 boys (70.71%) and 58 girls (29.29%) were included, and the average age of participants was 3.40 ± 1.06 years, with 3.33 ± 1.18 years in the TD group and 3.46 ± 0.95 years in the ASD group. The social adaptability of 107 ASD children was abnormal, including 37 children (34.5%) with marginal, 48 children (44.9%) with mild, 17 children (15.9%) with moderate, and 5 children (4.7%) with severe. In 91 TD children, there were 51 children (56.04%) with normal social adaptability, 38 children (41.75%) with marginal, 2 children (2.19%) with mild, and nobody with moderate or severe (Table 1).

**TABLE 1:** General information of the research objects.

Group	Age	Sex		Social adaptability				
			
		Male	Female	Normal	Marginal	Mild	Moderate	Severe
								
	(Mean ± SD)	*n* (%)	*n* (%)	*n* (%)	*n* (%)	*n* (%)	*n* (%)	*n* (%)
TD(*n* = 91)	3.33 ± 1.18	60 (65.93)	31 (34.07)	51 (56.04)	38 (41.75)	2 (2.19)	0 (0.00)	0 (0.00)
ASD(*n* = 107)	3.47 ± 0.96	80 (74.77)	27 (25.23)	0 (0.00)	37 (34.58)	48 (44.86)	17 (15.89)	5 (4.67)

### Comparison of developmental levels between children with autism spectrum disorders and typical development children

Compared with typically developing children, the ASD children had lower levels of gross motor, fine motor, language, adaptability, social ability, and developmental quotient than those of TD children, and the difference was statistically significant (Table 2).

**TABLE 2:** Results of comparison of developmental levels between autism spectrum disorders (ASD) and typical development (TD).

Group	DQ	Gross motor	Fine motor	Adaptability	Language	Social ability
						
	(Mean ± SD)	(Mean ± SD)	(Mean ± SD)	(Mean ± SD)	(Mean ± SD)	(Mean ± SD)
TD(*n* = 91)	89.20 ± 11.20	91.30 ± 12.70	82.23 ± 14.40	95.74 ± 11.90	90.52 ± 18.10	86.00 ± 17.01
ASD(*n* = 107)	58.42 ± 13.87	72.70 ± 14.89	52.52 ± 14.75	62.45 ± 17.17	48.71 ± 18.04	53.32 ± 15.94
*P*-value	< 0.001**	<0.001**	< 0.001**	<0.001**	< 0.001**	<0.001**

**P < 0.001.

### Association between motor development and social adaptability in children with autism spectrum disorders

In logistic regression, the dependent variable(*Y*) was the level of social adaptability. Due to the small sample size of children with severe social adaptability (five cases), this category was excluded from the regression. The “marginal” was used as the control group in the regression analysis. The logistic regression showed that after controlling for other variables, the regression coefficient value of fine motor was 0.807, *P* < 0.001, which means that fine motor has a significant positive relationship with social adaptability (Table 3). Parallel line test χ^2^ = 3.083, *P* = 0.687 > 0.05, which indicated that the parallel hypothesis was valid, that is, the regression equations were parallel to each other and could be analyzed by the ordered logistic process. So, the results of logistic regression showed that fine motor increased by 1 unit, and the OR value of one level decreased in social adaptability was 2.24 times (OR = e^0^.^807^ = 2.24).

**TABLE 3:** Regression analysis of motor development and social adaptability in children with autism spectrum disorders (ASD).

Model	Variable		Estimate	Std.Error	Wald	df	Sig.	95% Confidence interval	
							
								Lower bound	Upper bound
1	Threshold	Mild social adaptability	27.782	6.55	17.992	1	0.000*	14.945	40.619
		Moderate social adaptability	39.96	9.182	18.941	1	0.000*	21.964	57.957
	Location	Gross motor	0.027	0.03	0.792	1	0.373	–0.032	0.085
		Fine motor	0.66	0.151	19.066	1	0.000*	0.364	0.956
2	Threshold	Mild social adaptability	34.815	9.177	14.394	1	0.000*	16.829	52.801
		Moderate social adaptability	49.7	12.72	15.266	1	0.000*	24.769	74.631
	Location	Gross motor	0.09	0.065	1.935	1	0.164	–0.037	0.218
		Fine motor	0.807	0.211	14.628	1	0.000*	0.393	1.221
		Age	0.167	0.626	0.071	1	0.79	–1.059	1.393
		DQ	-0.183	0.205	0.797	1	0.372	–0.584	0.218
		Adaptability	0.12	0.087	1.933	1	0.164	–0.049	0.29
		Language	0.106	0.066	2.614	1	0.106	–0.023	0.235
		Social ability	–0.118	0.069	2.887	1	0.089	–0.254	0.018
		[Female = 0]	0.898	1.076	0.698	1	0.404	–1.21	3.007
		[Male = 1]	0^a^			0			

Link function: Logit. ^a^This parameter is set to zero because it is redundant. *P < 0.05.

## Discussion

In 1968, Kanner (27) first described the abnormal motor behaviors of infants with ASD, including clumsy gait and gross movement. Nearly 20 years, a growing number of studies confirmed that children with ASD have lesser action ability than TD children; show bulky action, fine motor and related action coordination. These abnormal behaviors can appear in the infant period (before the age of 2) (18), and obstacles in action ability development become more apparent as children with ASD grow (28). In this study, the movement scores of children with ASD were considerably lower than those of normal children. This finding is consistent with the results of Sally (29) on the motor skills of children with ASD aged 4–5 years. The development of fine motor and gross motor skills is the basis of children’s physical activity and daily learning life in growth. Motor development delay in children with ASD is less severe than that caused by simple motor development or common diseases. Moreover, the gross and fine motor skills of children with ASD may show temporary delay, and the children can eventually acquire the corresponding skills. Therefore, improvement in motor skills is also important in the intervention treatment of children with ASD.

Motor development defects are common in children with ASD and may further hinder the social adaptability of these children (29). The early motor development of children can be regarded as the early explicit intelligence of individuals, and the physical and mental development of individuals is mainly through the adaptation of motor to the environment (30). Because of motor development disorders, autistic children are less likely to participate in group activities, which limits their contact with peers and further affects the development of their social adaptability (31). Sipes (32) found a positive correlation between motor ability and social adaptation in children with ASD. This finding is consistent with the findings of the present study that children with low score on motor ability had similarly low scores on social adaptability, and vice versa. As far as current neuroscience is concerned, the cerebellum is believed to play important roles in controlling skilled movements and interacting with others in social environments (33). During exercise, in addition to the information from proprioceptors and visual and auditory organs transmitted to the cerebellum, the happy atmosphere of peer communication and group activities also stimulated the cerebellum and promoted the function of the cerebellum (34), which in turn led to the joint development of children’s motor ability and social adaptability. Mac Donald (35) found that children’s early activities and communication are supported by actions. Poor action ability makes children unable to participate in peer games and even weaken their social adaptability in the long run. Therefore, the improvement of children’s motor skills should also be the focus of early ASD intervention in addition to the improvement of children’s social adaptability.

In this study, as compared to gross motor skills, fine motor skills were found to be more strongly related to some social adaptability, which supports previous studies t in typically developing children (24, 36). As compared to gross motor skills, fine motor skills were found to be more strongly related to some social skills, which involve smaller muscles such as the hands and fingers, and are involved in activities like eating with utensils, finger-painting, cutting with scissors, and writing. Our motor skills play an important role in developing our cognition and social functioning, according to the theory of embodied cognition (37). Hellendoorn (38) found that fine motor functioning in children with ASD was related to visuospatial cognition, object exploration, and social orientation, as well as to language development, and fine motor functioning aids preschool students in interacting with both the physical and social environment and improves visuospatial cognition, which in turn increases language development. Fine motor skills are also important because they are highly correlated with social skills, and hand motor skills directly affect living ability. Difficulty fine motor skill affects the ability to take care of themselves, such as eating and dressing. In addition, children with low fine motor skills may not be able to participate in many social activities due to their inability to carry out motor functions necessary for play, which can lead to social estrangement (39). Furthermore, also found in the study of fine motor development in infants and children with ASD that fine motor is related to language development and that a worse fine motor development corresponds to a worse language development (40, 41). Evidence suggests that motor and social skills are interconnected at the neurophysiological level (24, 42). Early fine motor skills development and brain cognitive development overlap in time and space. Cognitive and motor skills are acquired in a basically similar way, and they are highly similar in the learning rate, learning effect, and learning stage. The smooth and effective development of early fine motor skills may be conducive to the maturation of early brain structure and function, and then promote the development of the cognitive system. The association between motor and social development in children with ASD may be explained by these transactions between brain structures. There may be consequences for multiple skill sets if there is dysfunction in one area of the brain (43). Furthermore, strengthening one or more of these skills early on might have positive effects elsewhere (44).

At present, the focus of intervention for children with autism is mainly on language and social interaction. And all interventions are aimed at improving the functional communication skills of children with ASD, such as opportunity learning, critical response training, TEACCH program and SCERTS mode (45). However, early motor skill intervention is often neglected. In fact, for any child, gross motor skill is more favorable for their own body control, and fine motor skill will be of great help to improve children’s cognition and social skills. Infants’ motor and cognitive development cannot be completely separated, especially the hand-eye coordination ability, which is an important indicator of cognitive development (46). The influence of fine motor is not only limited to social adaptation ability, but also has a great impact on learning ability and memory ability. As a special group, autistic children have their own characteristics in social adaptability and self-care. Strengthening the hand-eye coordination ability of autistic children through hand function training may play a certain role in improving the self-care ability of autistic children. Therefore, an early assessment and intervention of fine motor skills may be useful in improving the multiple abilities of children with ASD.

## Conclusion

This study found that in children with ASD, not only motor development is delayed, but also social adaptability is affected, and fine motor skill may be important for social adaptability. Motor problems can pose an additional burden on a child, affecting child’s quality of life and ability to interact socially. Thus, in future research, we can further clarify the possible role of fine motor development in improving social adaptability may help identify motor skills for early intervention, which may reduce the burden of ASD and may also help to provide optimal rehabilitation programs for these children and help them reach their full potential.

## Data availability statement

The raw data supporting the conclusions of this article will be made available by the authors, without undue reservation.

## Author contributions

YC, XF, HW, and AL conceived the study. YC and TW analyzed the data. XF wrote the manuscript. HL revised and refined the manuscript. NX, RS, and YW contributed to the collection of data. YC and XF was responsible for the integrity of the work as a whole. All authors critically reviewed various drafts of the manuscript and approved the final version.
